# Discovery of metabolic biomarkers for gestational diabetes mellitus in a Chinese population

**DOI:** 10.1186/s12986-021-00606-8

**Published:** 2021-08-21

**Authors:** Wenqian Lu, Mingjuan Luo, Xiangnan Fang, Rong Zhang, Shanshan Li, Mengyang Tang, Xiangtian Yu, Cheng Hu

**Affiliations:** 1 The Third School of Clinical Medicine, Southern Medical University, Guangzhou, China; 2 Department of Endocrinology and Metabolism, Fengxian Central Hospital Affiliated to the Southern Medical University, Shanghai, China; 3 Department of Endocrinology, University of Hong Kong Shenzhen Hospital, Shenzhen, China; 4 Shanghai Diabetes Institute, Shanghai Jiao Tong University Affiliated Sixth People’s Hospital, Shanghai, China; 5 Clinical Research Center, Shanghai Jiao Tong University Affiliated Sixth People’s Hospital, Shanghai, China; 6 Department of Endocrinology, First Affiliated Hospital of Gannan Medical University, Ganzhou, China

**Keywords:** Gestational diabetes mellitus, Metabolic biomarkers, Pregnancy, Pathogenesis

## Abstract

**Background:**

Gestational diabetes mellitus (GDM), one of the most common pregnancy complications, can lead to morbidity and mortality in both the mother and the infant. Metabolomics has provided new insights into the pathology of GDM and systemic analysis of GDM with metabolites is required for providing more clues for GDM diagnosis and mechanism research. This study aims to reveal metabolic differences between normal pregnant women and GDM patients in the second- and third-trimester stages and to confirm the clinical relevance of these new findings.

**Methods:**

Metabolites were quantitated with the serum samples of 200 healthy pregnant women and 200 GDM women in the second trimester, 199 normal controls, and 199 GDM patients in the third trimester. Both function and pathway analyses were applied to explore biological roles involved in the two sets of metabolites. Then the trimester stage-specific GDM metabolite biomarkers were identified by combining machine learning approaches, and the logistic regression models were constructed to evaluate predictive efficiency. Finally, the weighted gene co-expression network analysis method was used to further capture the associations between metabolite modules with biomarkers and clinical indices.

**Results:**

This study revealed that 57 differentially expressed metabolites (DEMs) were discovered in the second-trimester group, among which the most significant one was 3-methyl-2-oxovaleric acid. Similarly, 72 DEMs were found in the third-trimester group, and the most significant metabolites were ketoleucine and alpha-ketoisovaleric acid. These DEMs were mainly involved in the metabolism pathway of amino acids, fatty acids and bile acids. The logistic regression models for selected metabolite biomarkers achieved the area under the curve values of 0.807 and 0.81 for the second- and third-trimester groups. Furthermore, significant associations were found between DEMs/biomarkers and GDM-related indices.

**Conclusions:**

Metabolic differences between healthy pregnant women and GDM patients were found. Associations between biomarkers and clinical indices were also investigated, which may provide insights into pathology of GDM.

**Supplementary Information:**

The online version contains supplementary material available at 10.1186/s12986-021-00606-8.

## Introduction

Gestational diabetes mellitus (GDM), defined as diabetes diagnosed during pregnancy, affects approximately 15% of pregnant women globally [1]. Several risk factors are highly correlated with the development of GDM, including maternal obesity, advanced age, family history of diabetes mellitus, and history of abnormal glucose metabolism [2, 3]. Studies have found that GDM can lead to several short-term and long-term complications, including obesity, impaired glucose metabolism, and cardiovascular diseases, for both mothers and infants [4]. It was reported that in women with previous GDM the risk of developing diabetes after delivery was more than seven times that in women with normal glucose tolerance (NGT). For the offspring, they are more than two to eight times as likely to develop obesity, the metabolic syndrome, type 2 diabetes, and impaired insulin sensitivity and secretion [5]. Although the pathogenesis of GDM is still not well understood, metabolomics has introduced new insights into the pathology of GDM and has yielded potential biomarkers related to GDM [6].

Metabolomics is the comprehensive analysis of low molecular weight compounds, known as metabolites, in biological systems. As the end products of metabolic processes, metabolites can reflect the internal physiological status of the organism that changes in response to environmental factors. Metabolic profiling is a useful approach for qualitative and quantitative metabolite studies of cell, bio-fluids, and tissue [7]. In recent decades, metabolomics has been applied in various aspects, including disease diagnosis and treatment, metabolic pathway elucidation, biomarker discovery, and drug safety evaluation [8, 9]. For example, an untargeted metabolomics study has revealed a large number of pregnancy-related metabolic pathways and metabolites, five of which were able to predict gestational age in high accordance with ultrasound [10].

Many omics-based studies have been conducted for identifying biomarkers of GDM and for exploring the underlying mechanisms of its development [11, 12]. Previous proteomics studies have confirmed the downregulation of adiponectin among GDM women [13, 14]. Adiponectin could mediate antidiabetic metabolic effects through phosphorylation and activation of the 5'-AMP-activated protein kinase and acetyl coenzyme A carboxylase, thereby increasing fatty acid oxidation and glucose uptake in vivo. Conversely, downregulation of adiponectin may induce insulin resistance as well as GDM [15, 16]. Like proteomics, metabolomics could provide a deeper insight in the pathogenesis of GDM. Akturk et al. observed that asymmetric dimethylarginine was elevated in women with GDM during late pregnancy [17]. Cetin et al. found that in GDM pregnancies, valine, methionine, phenylalanine, isoleucine, leucine, ornithine, glutamate, proline, and alanine were increased while glutamine was significantly decreased [18]. Chen et al. revealed that 2-aminobutyric acid was associated with an increased likelihood of GDM in China [19]. Moreover, 26 serum metabolites investigated by Liu et al. contributed to GDM, including 1-methyladenosine, homovanillic acid sulfate, and glucosamine compared with healthy pregnant women. These identified biomarkers are involved in some metabolic pathways that mainly participate in lipid, carbohydrate, and amino acid metabolisms [20]. Although these investigations of metabolite profiles have identified branched-chain amino acids (BCAAs), aromatic amino acids, sulfur-containing amino acids, and other metabolites, their findings still lack consistency. Therefore, systemic analysis of the metabolites of GDM is necessary for obtaining more clues for GDM diagnosis and mechanism research.

In this study, we aimed to explore the metabolic difference between GDM women and normal pregnant women in the second and third trimester, using ultra-performance liquid chromatography coupled to tandem mass spectrometry (UPLC-MS/MS) system. Additionally, we expected to reveal the association of clinical indices with differentially expressed metabolites (DEMs) found in such metabolomics analysis.

## Materials and methods

### Study participant recruitment

All the samples were obtained from 200 healthy pregnant women and 200 GDM women in the second trimester, 199 normal controls, and 199 GDM patients in the third trimester at the University of Hong Kong-Shenzhen Hospital from 2016 to 2018. GDM patients were matched 1:1 with normal pregnant women. Matching was based on maternal age (± 3 years), pregestational BMI (± 3 kg/m^2^), and gestational week (± 3 weeks). Participants with cancer, kidney disease, heart disease, hepatic disease, other metabolic diseases or patients using medications that might affect glucolipid metabolism were excluded. The diagnostic criteria for GDM women were based on the standards recommended by the International Association of the Diabetes and Pregnancy Study Group. Glucose (75 g) was used for 2 h for conducting oral glucose tolerance test (OGTT). GDM was defined when fasting plasma glucose (FPG) was ≥ 5.1 mmol/L or 1-h plasma glucose was ≥ 10.0 mmol/L or 2-h plasma glucose was ≥ 8.5 mmol/L. All values for the OGTT less than the thresholds were considered normal. The study was approved by the ethics committee of the University of Hong Kong-Shenzhen Hospital ([2017]13). It was conducted according to relevant regulations, and informed consent was signed by every participant.

### Sample collection and serum metabolomics

Age, height, and weight were recorded for every participant. General background information, including family history of diabetes, reproductive history, and medical history, were collected. Information about pregnancy outcomes was acquired after delivery, from the maternal and infant medical records in the hospital. Body mass index (BMI) was calculated as body weight (in kg)/height squared (in m^2^). Serum samples obtained from 24 to the end of 27 gestational weeks were defined as the second-trimester group, while those after 28 gestational weeks were defined as the third-trimester group. Blood samples were drawn in the morning after an overnight fast through the antecubital vein. Glucose levels were measured using the hexokinase method on a Roche Cobas 701 analyzer (Roche, Ltd, Basel, Switzerland). HbA1c values were tested using an Arkray HA-8160 analyzer (Arkray, Ltd, Kyoto, Japan). Serum lipids, including total triglyceride (TG), total cholesterol (TC), high-density lipoprotein cholesterol (HDL-C), and low-density lipoprotein cholesterol (LDL-C) levels were measured with a Siemens ADVIA2400 fully automated chemistry analyzer (Siemens AG, Munich, Germany).

Metabolomic profiling was performed using Metabo-Profile (Shanghai, China). Serum samples were stored at − 80 °C until analysis. All of the standards of targeted metabolites were accurately weighed and prepared in water or methanol to obtain individual stock solution with a concentration of 5 mg/mL. Appropriate amount of each stock solution was mixed to create stock calibration solutions. A mixture of stable isotope labeled internal standards were prepared in methanol at a concentration of 50 µM/L. 25 µL of serum was added to a 96-well plate and the plate was transferred to the Biomek 4000 automation workstation (Biomek 4000, Beckman Coulter, Inc., California, USA). Approximately 120 µL of ice-cold methanol with partial internal standards was automatically added to each sample for extracting the metabolites. After vortexing for 5 min, the mixture was centrifuged for 30 min at 4000 g; 30 µL of supernatant was transferred to a new 96-well plate, and 20 µL of freshly prepared derivative reagents (3-Nitrophenylhydrazine) was added to each well. After derivatization for 60 min at 30 °C, 330 µL of ice-cold 50% methanol solution was added for dilution. The samples were stored at − 20 °C for 20 min and were centrifuged at 4000 g for 30 min at 4 °C. Approximately 135 µL of supernatant was mixed with 10 µL of internal standards in each well of a new 96-well plate. Serial dilutions of derivatized stock standards were added to the left wells, and the plate was ready for analysis.

Chromatographic separation was performed on an ACQUITY UPLC BEH C18 VanGuard pre-column (2.1×5 mm, 1.7 µm) and an ACQUITY UPLC BEH C18 analytical column (2.1×100 mm, 1.7 µm). The temperature of the column and the sample manager was set at 40 °C and 10 °C, respectively. The mobile phase A was water with 0.1% formic acid, while B was a mixture of acetonitrile and isopropanol (70:30). Gradient conditions were 0–1 min, 5% B; 1–11 min, 5%–78% B; 11–13.5 min, 78%–95% B; 13.5–14 min, 95%–100% B; 14–16 min, 100% B; 16–16.1 min, 100%–5% B; 16.1–18 min, 5% B. The flow rate was 0.4 mL/min with a 5 µL injection volume.

**Fig. 1:**
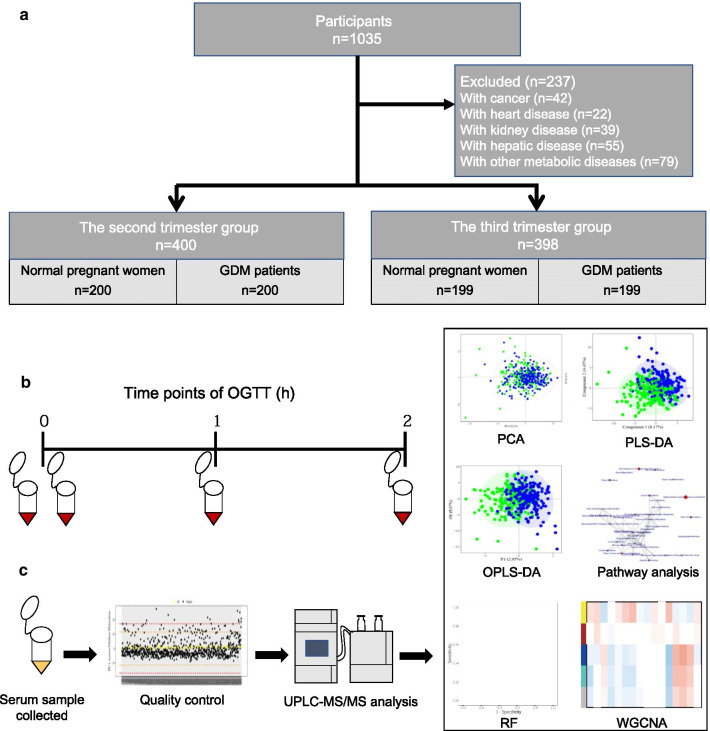
The design and analysis workflow of the study. **a** Participant selection. **b** Time points when blood samples were collected during OGTT. One blood sample at the zero-time point was obtained before OGTT for the serum sample. **c** Metabolic analysis pipeline

The mass spectrometer was operated in positive electrospray ionization (ESI +) mode with a capillary voltage of 1.5 kV as well as the negative electrospray ionization (ESI −) mode with a capillary voltage of 2 kV. The temperature of the ion source and desolvation was 150 °C and 550 °C, respectively. The desolvation gas flow was set at 1000 L/h. Raw data files generated by UPLC-MS/MS were processed using Masslynx software (v4.1, Waters, Milford, MA, USA) for performing peak integration, calibration, and quantitation for each metabolite. An overview of the research design and analysis workflow is presented in Fig. 1.

### Bioinformatics analysis and metabolic biomarker recognition

For the analysis of clinical characteristics, participants were divided into four groups: the second-trimester GDM group (group 1), second-trimester normal group (group 2), third-trimester GDM group (group 3), and third-trimester normal group (group 4). Clinical characteristics between the same gestational-stage groups (group1 vs. group 2 and group3 vs. group 4) were compared using the Student’s paired *t*-test or signed-rank test for continuous variables and chi-square tests for categorical variables with SAS 9.3 (SAS Institute, Cary, NC, USA). After normality testing for continuous variables, the data was presented as mean ± standard deviation (SD) or median (interquartile range). The level of significance was set at *p* < 0.05.

To observe changes in metabolites between the same gestational-stage groups, the univariate and multivariate analyses were conducted, including differential expression analysis using *t*-test, fold-change with volcano plot, principal component analysis (PCA), partial least square discriminant analysis (PLS-DA), and orthogonal partial least square discriminant analysis (OPLS-DA). Data processing was performed using the iMAP platform (v1.0, Metabo-Profile, Shanghai, China). *p* < 0.05 and |log2FC|> 0 in the univariate analysis and variable importance in the projection (VIP) > 1 in the multivariate analysis were the standards for screening DEMs. To investigate the biological meaning of DEMs, enrichment analysis was performed with the small molecule pathway database (SMPDB) with *p* < 0.05 regarded as the significance level, and pathway analysis was also performed using *p* values to account for significance and false discovery. A threshold of *p* < 0.05 was used for considering the function or pathway to be impactful. Metabolites were further analyzed using random forest (RF) for identifying potential biomarkers based on which logistic regression (LR) models were constructed for evaluating predictive efficiency. Area under the curve (AUC) for receiver operating characteristic (ROC) curves were calculated for assessing the performance of potential biomarkers with LR for GDM. The determination of sensitivity and specificity at the optimal cut-off point was defined by the minimum distance to the top-left corner. Longitudinal analysis for catching continue-changed metabolic biomarkers with the disease was performed by extracting the shared candidate metabolic biomarkers from above classification-model evaluated biomarkers in two pregnancy stages. The weighted gene co-expression network analysis (WGCNA) [21] was used for inferring the association between metabolite modules and clinical indices.

## Results

### Clinical characteristic of study participants

The clinical characteristics of all participants are presented in Table 1. Compared with normal pregnant women, pregnant women who developed GDM had higher FPG, 1-h, and 2-h blood glucose levels after an OGTT and smaller changes in BMI during pregnancy, in both the second and third trimesters. HbA1c, TG, TC, and LDL were statistically different between the case and control groups in the second trimester, which were consistent with previous research conclusions [22, 23]. TC, delivery gestational week, neonatal weight, and neonatal length were statistically different between GDM patients and healthy pregnant women in the third trimester. Women with GDM were more likely to have a family history of diabetes. Age, BMI, and gestational week were similar between the two groups in each trimester.

**Table 1:** Characteristics of the NGT and GDM groups in the second and third trimesters

	Subjects in the second trimester			Subjects in the third trimester		
	NGT (n = 200)	GDM (n = 200)	P	NGT (n = 199)	GDM (n = 199)	P
Age (years)	29 (27,30)	29 (27,30)	0.117	31 (29,34)	31 (29,35)	0.208
Pregestational BMI (kg/m^2^)	20.33 ± 2.58	20.72 ± 2.77	0.151	21.33 ± 2.26	21.44 ± 2.39	0.665
Changes of BMI (kg/m^2^)	5.26 ± 1.31	4.63 ± 1.47	< 0.001	5.6 ± 1.46	4.68 ± 1.61	< 0.001
Gestational age (week)	25.71 (24.86, 26.43)	25.86 (25, 26.5)	0.925	29 (28, 31.1)	29.29 (28, 31.1)	0.395
FBG (mmol/L)	4.4 ± 0.32	4.56 ± 0.38	< 0.001	4.49 ± 0.25	4.63 ± 0.33	< 0.001
1 h-PG (mmol/L)	7.22 ± 1.29	9.67 ± 1.34	< 0.001	7.35 ± 1.37	9.94 ± 1.51	< 0.001
2 h-PG (mmol/L)	6.27 ± 0.92	8.5 ± 1.35	< 0.001	6.44 ± 1.06	8.79 ± 1.56	< 0.001
HbA1c			< 0.001			0.962
mmol/mol	32.47 ± 2.08	33.45 ± 2.53		32.93 ± 3.52	32.94 ± 3.14	
%	5.12 ± 0.19	5.21 ± 0.23		5.16 ± 0.32	5.16 ± 0.29	
Total cholesterol (mmol/L)	6.13 ± 1.05	5.83 ± 1.11	0.005	6.64 ± 1.22	6.13 ± 1.16	< 0.001
Triglycerides (mmol/L)	1.93 ± 0.65	2.33 ± 0.86	< 0.001	2.91 ± 1.38	2.94 ± 1.78	0.850
LDL-C (mmol/L)	2.91 ± 0.74	3.24 ± 0.96	< 0.001	3.29 ± 0.89	3.40 ± 0.91	0.233
HDL-C (mmol/L)	2.05 ± 0.37	2.02 ± 0.42	0.488	1.98 ± 0.39	1.95 ± 0.40	0.395
Delivery gestational age (week)	39 (38,40)	39 (38,40)	0.120	39 (39,40)	39 (38,40)	0.004
Neonatal weight (kg)	3.29 ± 0.38	3.23 ± 0.46	0.127	3.38 ± 0.39	3.26 ± 0.41	0.005
Neonatal length (cm)	50 (50,50)	50 (50,50)	0.214	50 (50,51)	50 (50,50)	0.010
Family history of diabetes, n (%)	5(2.5)	48(24)	< 0.001	27(13.6)	53(26.6)	0.001

Data are presented as means ± SD, median (interquartile range) or n (%)

*NGT* normal glucose tolerance, *GDM* gestational diabetes mellitus, *FBG* fasting blood glucose, *1 h-PG* one hour postprandial glucose, *2 h-PG* two hours postprandial glucose, *LDL-C* low-density lipoprotein cholesterol, *HDL-C* high-density lipoprotein cholesterol

### Metabolomics profiling of study participants

**Fig. 2:**
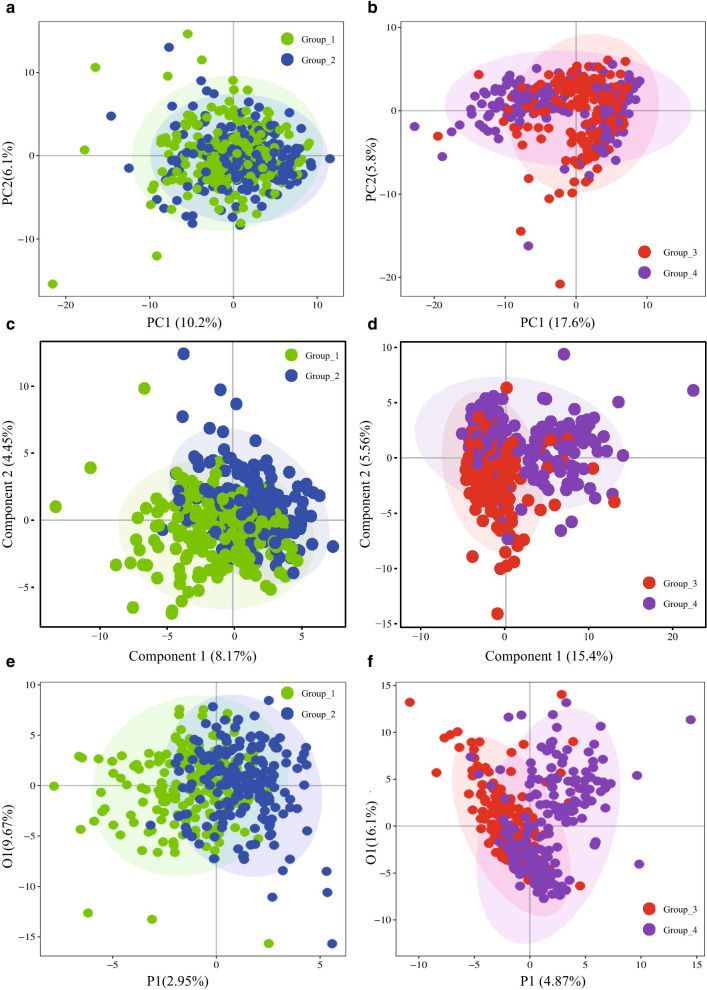
Discrimination analysis of metabolomic profiles. **a** PCA model in the second trimester. **b** PCA model in the third trimester. **c** PLS-DA model in the second trimester. **d** PLS-DA model in the third trimester. **e** OPLS-DA model in the second trimester. **f** OPLS-DA model in the third trimester. Blue refers to the control group while green refers to the case group in the second trimester, and purple refers to normal pregnant women while red refers to GDM patients in the third trimester

**Fig. 3:**
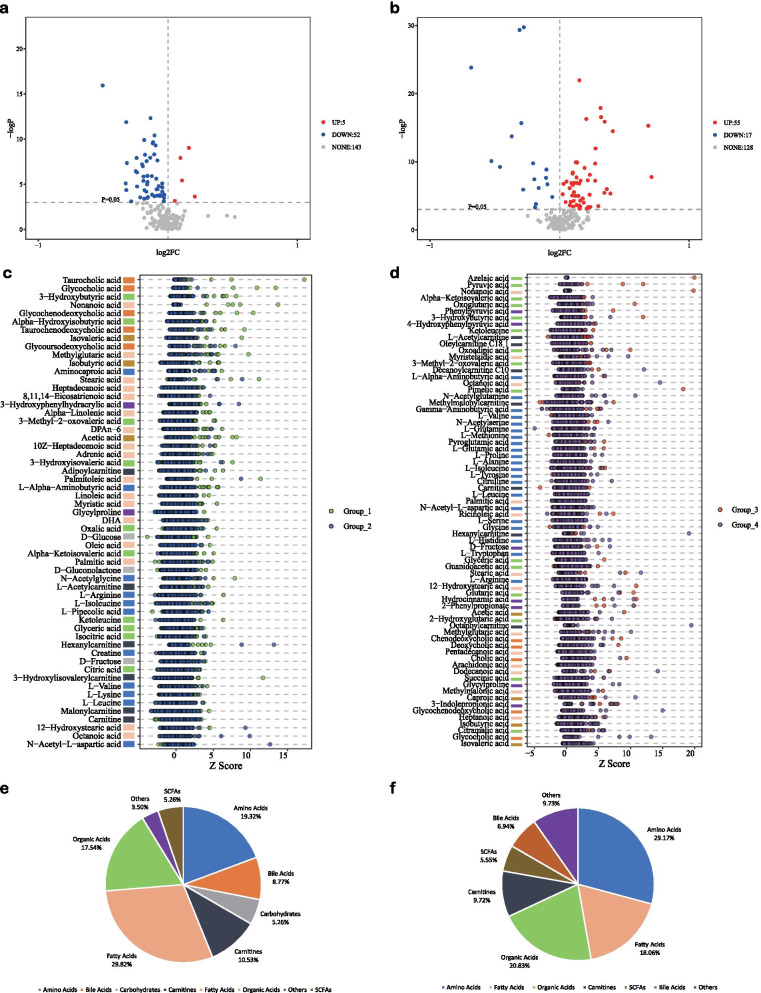
Differential expression analysis of metabolomic profiles. **a** Volcano plot of DEMs in the second-trimester group. **b** Volcano plot of DEMs in the third-trimester group. **c** Test statistics of DEMs in the second-trimester group. **d** Test statistics of DEMs in the third-trimester group. **e** Classification of DEMs in the second-trimester group. **f** Classification of DEMs in the third-trimester group

As shown in the metabolomics profiles (Fig. 2a, b), the case group (GDM) did not separate from the control group (normal) in the second and third trimester with PCA; moreover, PLS-DA showed a relatively clear discrimination for both the trimesters (Fig. 2c, d). Finally, the results of OPLS-DA (Fig. 2e, f) indicated the possibility of evaluating the differences between GDM patients and normal controls with metabolite abundance. The results showed that R^2^ and Q^2^ of the OPLS-DA model in the second trimester were 0.347 and 0.165, while in the third trimester were 0.324 and 0.201, respectively. Permutation tests (n = 200) were employed for validating the predictive ability of the built OPLS-DA models (Additional file 1: Figure S1). The R^2^ and Q^2^ values derived from the permuted data were lower than the original values, which demonstrated that the OPLS-DA model did not overfit. The VIP values of the OPLS-DA model and criteria, including |log2FC|> 0 with *p* < 0.05, were further employed for determining the DEMs. A volcano plot provided a quick way to display the differences in metabolite expression levels between normal pregnant women and GDM patients with statistical significance (Fig. 3). As shown in the volcano plot, of 200 metabolites found in this study, 57 metabolites in the second-trimester group (Additional file 4: Table S1) and 72 metabolites in the third-trimester group (Additional file 5: Table S2) were considered DEMs. The top five DEMs in the second-trimester group were 3-methyl-2-oxovaleric acid, 3-hydroxybutyric acid, palmitic acid, alpha-hydroxyisobutyric acid, and acetic acid. As for the third-trimester group, they were ketoleucine, alpha-ketoisovaleric acid, pyruvic acid, L-tryptophan, and succinic acid. Most DEMs were down-regulated during the second trimester (Fig. 3a), while many DEMs were positively-regulated during the third trimester (Fig. 3b), suggesting different metabolomic dysfunction at different trimester stages. Amino acids, fatty acids, and organic acids account for around 70% of DEMs found in the second- and third-trimester stages (Fig. 3e, f).

### Metabolic enrichment of biological function and pathway relevant to GDM

**Fig. 4:**
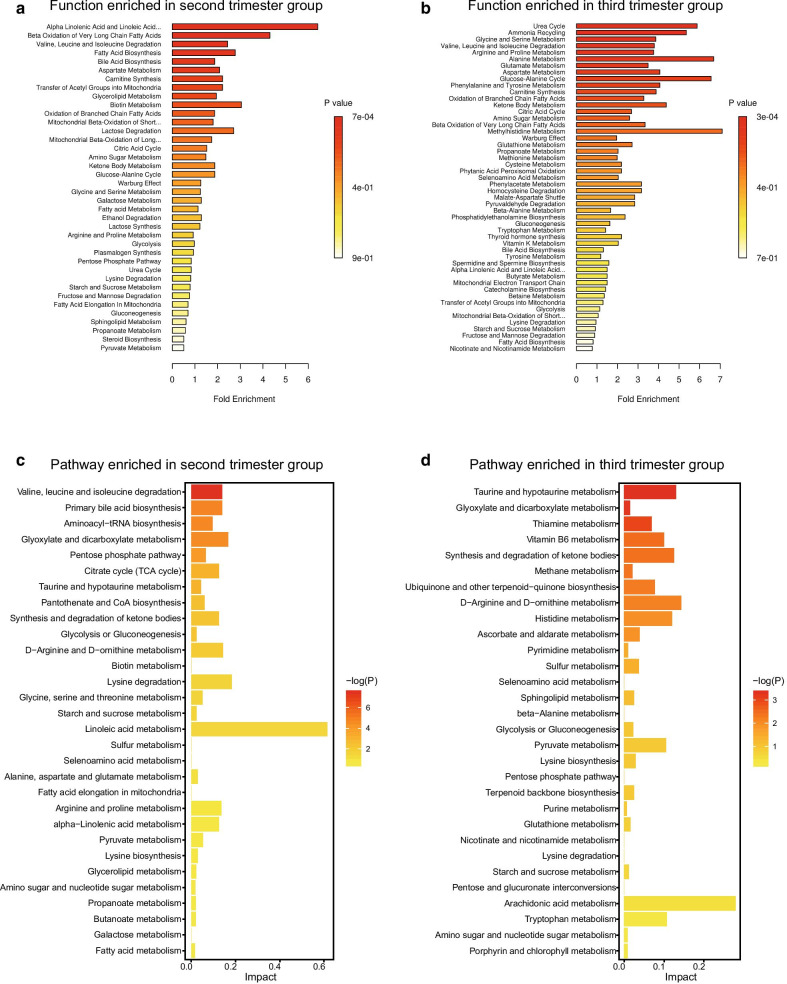
The enrichment analysis of metabolomic profiles. **a** Function enrichment for DEMs in the second-trimester group. **b** Function enrichment for DEMs in the third-trimester group. **c** Pathway enrichment for DEMs in the second- trimester group. **d** Pathway enrichment for DEMs in the third-trimester group

The DEMs for each comparison group were evaluated using enrichment analysis with SMPDB (Fig. 4a, b). In the second-trimester group, the alpha linolenic acid and linoleic acid metabolism pathways had the highest fold enrichment, lowest *p* value (*p* < 0.001), and FDR of < 0.1. Other significant functions included beta oxidation of very long chain fatty acids and valine-leucine-isoleucine degradation (Fig. 4a and Table 2, Additional file 6: Table S3). In the third-trimester group, functions such as urea cycle, ammonia recycling, glycine and serine metabolism, valine-leucine-isoleucine degradation, arginine and proline metabolism, alanine metabolism, glutamate metabolism, aspartate metabolism, glucose-alanine cycle, phenylalanine and tyrosine metabolism, and carnitine synthesis were significantly associated with the corresponding DEMs (Fig. 4b and Table 3, Additional file 7: Table S4).

**Table 2:** Enrichment analysis of the second trimester group

Pathway associated metabolite sets	Enriched compounds	Total	Hits	Raw *p*	Holm *p*	FDR
Alpha linolenic acid and linoleic acid metabolism	Linoleic acid; alpha-linolenic acid; docosahexaenoic acid; adrenic acid; 8,11,14-eicosatrienoic acid	19	5	0.000693	0.0679	0.0679
Beta oxidation of very long chain fatty acids	L-carnitine; L-acetylcarnitine; Caprylic acid	17	3	0.0292	1	1
Valine-leucine-isoleucine degradation	Alpha-ketoisovaleric acid; L-Valine; L-Isoleucine;3-Methyl-2-oxovaleric acid; L-Leucine; Ketoleucine;	60	6	0.0314	1	1

Pathway associated metabolite sets with* p* value < 0.05 are shown in the table. Total means total number of metabolites in the metabolite set; Hits mean number of metabolites from GDM patients in the metabolite set; Raw *p* refers to original *p* value in the enrichment analysis; Holm *p* refers to adjusted raw *p* value by Holm-Bonferroni method; FDR is the false discovery rate

**Table 3:** Enrichment analysis of the third trimester group

Pathway associated metabolite sets	Enriched compounds	Total	Hits	Raw *p*	Holm *p*	FDR
Urea cycle	Pyruvic acid; Oxoglutaric acid; L-Alanine; L-Arginine;L-Glutamine Citrulline	29	6	0.000325	0.0318	0.0176
Ammonia recycling	Glycine; L-Histidine; L-Glutamine; L-Serine; Oxoglutaric acid; Pyruvic acid	32	6	0.000574	0.0557	0.0176
Glycine and serine metabolism	Glycine;Guanidoacetic acid;Pyruvic acid;L-Alanine;L-Serine;L-Arginine Oxoglutaric acid; L-Methionine	59	8	0.000638	0.0612	0.0176
Valine-leucine-isoleucine degradation	Alpha-ketoisovaleric acid; L-Valine; Methylmalonic acid; Oxoglutaric acid; Succinic acid; Ketoleucine; 3-Methyl-2-oxovaleric acid; L- Leucine	60	8	0.000718	0.0682	0.0176
Arginine and proline metabolism	Glycine; Guanidoacetic acid; L- Proline; Oxoglutaric acid; Succinic acid; L-Arginine; Citrulline	53	7	0.00173	0.163	0.034
Alanine metabolism	Glycine; Oxoglutaric acid; Pyruvic acid; L-Alanine	17	4	0.00221	0.206	0.0362
Glutamate metabolism	Glycine; L-Alanine; Oxoglutaric acid; Pyruvic acid; Succinic acid; L-Glutamine	49	6	0.00571	0.525	0.0747
Aspartate metabolism	Oxoglutaric acid; L-Arginine; L- Glutamine; N-Acetyl-L-aspartic acid; Citrulline	35	5	0.00609	0.555	0.0747
Glucose-alanine cycle	L-Alanine; Oxoglutaric acid; Pyruvic acid	13	3	0.00896	0.807	0.0976
Phenylalanine and tyrosine Metabolism	L-Tyrosine; Phenylpyruvic acid; 4-Hydroxyphenylpyruvic acid; Oxoglutaric acid	28	4	0.0144	1	0.141
Carnitine synthesis	Glycine; Oxoglutaric acid; Succinic acid	22	3	0.0388	1	0.346

Pathway associated metabolite sets with* p* value < 0.05 are shown in the table

Pathway analysis was also performed for investigating the function of DEMs. 32 pathways were observed, nine of which were significantly enriched in the second-trimester group (Fig. 4c, Additional file 8: Table S5), among which two pathways, valine-leucine-isoleucine biosynthesis and valine-leucine-isoleucine degradation, played key roles in reflecting the changes in metabolites. For the third-trimester group, 48 pathways were found, of which 21 were significantly enriched with DEMs (Fig. 4d, Additional file 9: Table S6).

Furthermore, remarkable differences also exist between different trimester stages on function and pathway levels (Fig. 4 and Additional file 2: Fig. S2), suggesting that stage-specific biomarkers and diagnostic models should be considered.

### Selection of potential metabolic biomarkers for GDM

After observing metabolomics differences between the groups and reliable functional enrichment analysis, it was necessary to establish a diagnostic model for predicting the presence of GDM in pregnant women and for selecting potential metabolite characteristics with the importance determined using machine learning algorithms [24, 25].

**Fig. 5:**
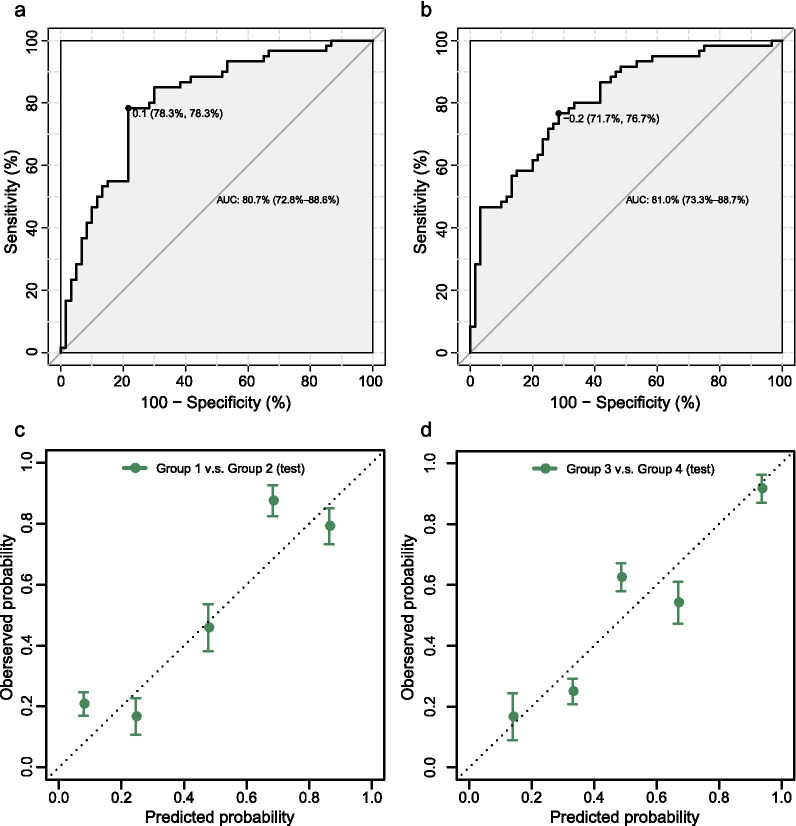
Validation ROC curves of candidate metabolite biomarkers. **a** ROC curve in the second-trimester group. **b** ROC curve in the third-trimester group. **c** Calibration plot of the second-trimester group. **d** Calibration plot of the third-trimester group. X-axis shows average predicted probability values for each decile, and y-axis shows corresponding observed probability in each decile. Error bars represent 95% confidence intervals of mean predicted probabilities

For the second-trimester group, samples were first divided into training data with 70% samples and test data with 30% samples. The RF model was then learned on the training data for obtaining the importance score for each metabolite, based on which the candidate metabolite biomarkers were selected with top large importance scores and with overlaps to DEMs as much as possible. Next, the LR model was constructed on the training data with such metabolite biomarkers (Additional file 10: Table S7), with a training AUC of 0.808. Finally, the second-trimester group-specific LR model was validated on the test data and achieved a testing AUC of 0.807 (Fig. 5a). Similarly, for the third-trimester group, the metabolite biomarkers (Additional file 10: Table S7) were selected using RF, and an LR model was built, which had learning performance as AUC 0.819 and validation performance as AUC 0.810 on the test data (Fig. 5b). In practice, patients diagnosed as positive by our new diagnostic method have about 30% probability of actual disease when the prevalence of GDM is 11.91% [26]. The positive predictive value in the second-trimester group is 0.328, and the negative predictive value is 0.964 (when sensitivity and specificity are all 0.783, the best diagnostic bounds), while the positive predictive value in the third-trimester group is 0.269, and the negative predictive value is 0.958 (when sensitivity is 0.767 and specificity is 0.717). In addition, almost all identified biomarker candidates were DEMs (Table 4). Of note, there are three common potential metabolic biomarkers (3-hydroxybutyric acid, isobutyric acid, and isovaleric acid) discovered both in second- and third-trimester groups, whose trend changes longitudinally across pregnancy were displayed in Additional file 3: Figure S3. As the pregnancy progressed, these three metabolic biomarkers significantly increased among normal pregnant women, while in GDM patients only 3-hydroxybutyric acid was increased. We also assess the goodness of fit between the predicted and real probabilities with calibration plot (Fig. 5c, d), evaluating how similar the predicted probability values are to the actual probabilities.

**Table 4:** Candidate metabolite biomarkers associated with GDM in the second and third trimester

Name	Second trimester	Third trimester
3-Methyl-2-oxovaleric acid	Grey*	Grey*
D-Gluconolactone	Grey*	
D-Glucose	Blue*	
3-Hydroxybutyric acid	Grey*	
Alpha-Hydroxyisobutyric acid	Grey*	
Isobutyric acid	Grey*	Turquoise*
Isovaleric acid	Grey*	Turquoise*
Octanoic acid	Brown*	
Glycocholic acid	Grey*	
Nonanoic acid	Grey*	
Myristic acid	Turquoise*	
DHA	Turquoise*	
Palmitic acid	Turquoise*	
Glycylproline		Turquoise*
Alpha-Ketoisovaleric acid		Grey*
Ketoleucine		Grey*
Acetic acid		Grey*
Caproic acid		Turquoise*
Heptanoic acid		Turquoise*
Pyruvic acid		Yellow*
Arachidonic acid		Blue*
Adrenic acid		Blue
Citramalic acid		Turquoise*

### Clinical relevance of metabolic biomarkers for GDM

**Fig. 6:**
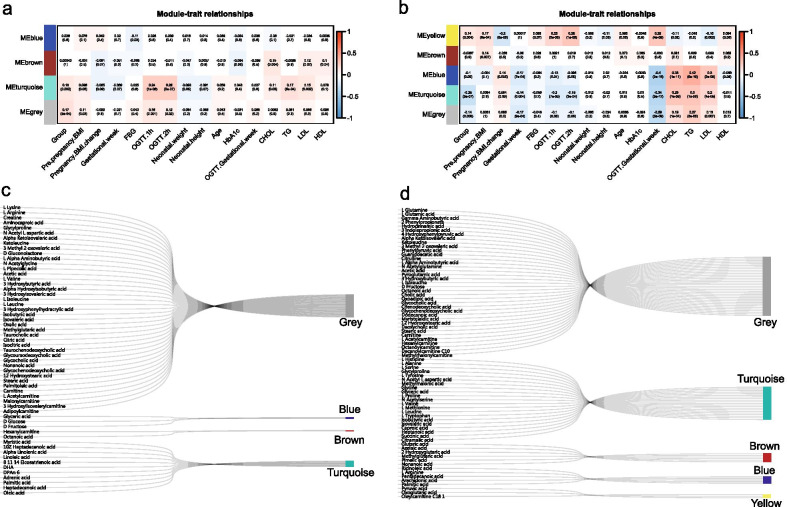
Clinical relevance of DEMs related to GDM. **a** The WGCNA for metabolite module and clinical indices in the second-trimester group. **b** The WGCNA for metabolite module and clinical indices in the third-trimester group. **c** DEMs’ distribution in metabolite modules in the second-trimester group. **d** DEMs’ distribution in metabolite modules in the third-trimester group

Finally, to confirm the clinical relevance of metabolic biomarkers associated with GDM, WGCNA was performed for inferring the association between metabolite modules and clinical indices. As shown in Fig. 6a, four modules were detected for the second-trimester group. Module turquoise was significantly associated with GDM (i.e., group index) and many other important clinical indices, including pre-pregnancy BMI, OGTT, TC, TG, and LDL. Additionally, this module significantly included 11 DEMs (P = 9.99e^−04^), compared to module blue containing three DEMs (P = 0.666), module brown containing two DEMs (P = 0.580), and module grey containing 41 DEMs (P = 0.868) (Fig. 6c). Similarly, five modules were found for the third-trimester group (Fig. 6b), where the module turquoise contains 21 DEMs (P = 6.737e^−08^), module blue contains five DEMs (P = 0.944), module brown contains six DEMs (P = 0.090), module grey contains 37 DEMs (P = 0.992), and module yellow contains three DEMs (P = 0.606) (Fig. 6d). At this time, module turquoise was associated with group, OGTT, TC, TG, and LDL; and module yellow was positively associated with the group, pre-pregnancy BMI, and OGTT. It should be noted that the biomarker candidates are almost DEMs (Table 4); thus, they have similar contribution to particular modules and their associations with GDM.

## Discussion

Using UPLC-MS/MS for metabolomics analysis, detailed information was obtained on the metabolic changes in normal pregnant women and GDM patients in our study. The changes in serum metabolites were further investigated using univariate and multivariate statistical analyses. Thirteen candidate metabolite biomarkers found in the second-trimester group and thirteen found in the third-trimester group contributed to GDM when compared with healthy pregnant women. According to metabolic enrichment and pathway analyses, valine-leucine-isoleucine degradation in the second-trimester group was consistently found in both analyses. In the third trimester, valine-leucine-isoleucine degradation and glycine, serine, arginine, proline, alanine, glutamate, aspartate, and phenylalanine metabolisms were consistent in both the analyses. Metabolic biomarkers have been found by RF, and LR models based on which showed high predictive efficiency. Furthermore, these biomarkers demonstrate remarkable relationship with clinical indices.

BCAAs, which consist of valine, leucine, and isoleucine, were higher in the GDM group than in the NGT group in our study. Although some studies have found that the levels of BCAAs did not differ significantly between GDM and NGT [27, 28], several studies have shown that elevated BCAAs in GDM patients might serve as biomarkers for GDM [29–31]. Phosphatidylinositol-3-kinase (PI3K)/ protein kinase B (Akt)/mammalian target of rapamycin (mTOR) signaling is involved in functions such as protein and lipid biosynthesis [32]. Mammalian target of rapamycin complex 1 (mTORC1) is controlled by the small GTPase Ras homolog enriched in brain (Rheb) and tuberous sclerosis complex (TSC) complex (TSC1 and TSC2) [33]. When insulin exists, it activates insulin receptor and insulin receptor substrate (IRS), which further promotes the activation of PI3K and Akt. Akt inhibits the TSC complex, allowing Rheb to stimulate mTORC1 to mediate cellular functions [34]. Ribosomal S6 protein kinase 1 (S6K1), one of the mTORC1 substrate, participates in the Rheb/mTOR/S6K pathway. However, constitutive activation of the Rheb/mTOR/S6K pathway can induce a negative feedback and thus cause insulin resistance [35]. BCAAs might be associated with insulin resistance in GDM women [36, 37]. One potential mechanism is that elevated BCAAs levels lead to activation of the mTOR/S6K1 pathway and serine phosphorylation of IRS1, contributing to inhibition of insulin-induced PI3K activation and insulin resistance [38]. Also, BCAAs dysmetabolism could result in the accumulation of toxic BCAA metabolites that cause β-cell mitochondrial dysfunction and highly susceptibility to insulin resistance [39].

In our study, glycine and serine metabolisms were lower in the GDM group, which is consistent with findings of previous studies. Takashina et al. observed that fasting glucose and 2-h plasma glucose levels or the homeostasis model assessment of insulin resistance negatively correlated with glycine levels, and the homeostasis model assessment for the β-cell function index negatively correlated with glycine and serine levels [40]. Moreover, oral glycine has been reported to increase insulin secretion without affecting insulin sensitivity [41].

Additionally, arginine and proline levels were also lower in the GDM group. It has been reported that arginine and its metabolites promote insulin secretion [42] and improve insulin resistance in humans [43]. Arginine plays multiple beneficial roles against metabolic abnormalities, but it might also induce oxidative stress [44]. Proline is absorbed and metabolized into glutamine, which may enter the tricarboxylic cycle and ultimately be converted into glucose. One study demonstrated that the ingestion of proline with glucose attenuated the glucose area response without affecting insulin response and decreased glucagon levels compared to glucose alone [45].

Alpha linolenic acid is a precursor of polyunsaturated fatty acids, which mainly contain omega-6 and omega-3 fatty acids. A meta-analysis by Zhong et al. investigated the efficacy of omega-3 fatty acid for GDM, revealing that omega-3 fatty acids supplementation in GDM patients could reduce FPG and HOMA-IR score [46]. Omega-3 fatty acids were involved in the mechanism of increasing β-oxidation of fatty acids, improving antioxidant functions and insulin action, and reducing lipogenesis [47].

Short-chain fatty acids (SCFAs) refer to those fatty acids that have one to six carbons. Previous study has indicated that increased expression of free fatty acid receptor-2 and alteration of its endogenous ligands SCFAs contributed to glucose homeostasis by improving insulin secretion throughout gestation [48]. SCFAs also diminished late gestational androgen excess through suppression of adenosine deaminase /xanthine oxidase pathway, which protected against glucose dysmetabolism and poor fetal outcome [49]. SCFAs can not only impact metabolism, but also influence cardiovascular diseases. SCFAs are recognized by G protein-coupled receptors, one of which is G protein-coupled receptor 41(Gpr41). Another SCFA receptor olfactory receptor 78, together with Gpr41, participated in the modulation of blood pressure. While Gpr41 lowered baseline blood pressure [50, 51], olfactory receptor 78 induced the hypertensive effect of SCFAs [51]. Acetate, one of major SCFAs, decreases among GDM patients in our study. It was believed to enhance glucose incorporation and lipid metabolism through activating AMP-activated protein kinase [52, 53]. Additionally, supplementation of acetate significantly increased the number of activated Treg cells [54], which could attenuate cardiac hypertrophy and fibrosis and improve electric remodeling in hypertension/Angiotensin II–induced cardiac damage [55].

Of note, 3-hydroxybutyric acid was selected as a potential metabolic biomarker in both the trimester groups. As a classic ketone body, the levels of 3-hydroxybutyric acid increases because of the oxidation of free fatty acids and excess acetyl-CoA. ATP production from fatty acids and carbohydrate oxidation happens out of control, resulting in increased acetyl-CoA levels. A study in diabetic rats showed that inefficient utilization and mobilization of glucose may contribute to the elevation of 3-hydroxybutyric acid levels [56].

In the WGCNA, carnitine was included in the module grey for both the trimesters. Previous studies have shown that using 2 g/day of L-carnitine resulted in a reduction of TC and LDL, and its mechanism may be related to the phenomena of insulin resistance and lipotoxicity [57]. However, a higher dose of L-carnitine had different effects, since it contributed to the elevation of TG, apolipoprotein-A1, and apolipoprotein-B100 levels [58]. While BCAAs are in the module grey in the second-trimester group, isoleucine belongs to the module grey, leucine and valine are in the module turquoise for the third-trimester group. It is widely accepted that BCAAs transaminase helps in the conversion of isoleucine and valine into branched-chain α-ketoacids, which are further transformed into propinonyl-CoA by the branched-chain α-ketoacid dehydrogenase complex. Propinonyl-CoA can become methylmalonyl-CoA with relevant carboxylase. Methylmalonyl-CoA mutase (MUT) is an enzyme that catalyzes the conversion of methylmalonyl-CoA to succinyl-CoA [59]. Based on experiments with mice, decreased *Mut* expression led to higher body weight, hyperinsulinemia, elevated fasting glucose and increased triglyceride [60].

Actually, this study has some limitations. First, in a cross-sectional study design, metabolites were only measured at one point; thus, further prospective cohort studies are needed for establishing the dynamic association of these metabolites with GDM. Second, though metabolite biomarkers in the first trimester allow early diagnosis and timely intervention, we mainly focused on the metabolic changes during the second and third trimesters due to lack of enough participants recruited in the first trimester stage. Third, while the second trimester starts at week 14 of pregnancy and lasts through the end of week 27, participants were recruited from 24 weeks to the end of 27 gestational weeks for the second-trimester group; thus, important information may have missed out. Fourth, the precise molecular mechanisms underlying the development of GDM remain unclear and mechanistic studies need to be conducted for clarifying the exact roles of these discovered metabolites in GDM.

## Conclusion

In conclusion, with the analysis of serum samples, our study suggested that specific metabolomic profile existed among GDM patients. Several key metabolites, such as glycine, serine, proline, and 3-hydroxybutyric acid, are associated with GDM in the second or third trimester, and potential biomarkers for GDM have been identified. These metabolites mainly participated in fatty acid and amino acid metabolism, which may shed light on the pathology of GDM. However, further research is needed to confirm our findings and explore the underlying molecular mechanisms.

## Supplementary Information

**Additional file 1**.** Figure S1**: Permutation test in the second trimester group (a) and the third trimester group (b).

**Additional file 2**.** Figure S2**: Overlap statistic of different findings in two trimester groups.

**Additional file 3**.** Figure S3**: Longitudinal changes of three potential metabolic biomarkers. *P < 0.05.

**Additional file 4**.** Table S1**: List of DEMs in the second trimester group.

**Additional file 5**.** Table S2**: List of DEMs in the third trimester group.

**Additional file 6**.** Table S3**. Function enrichments of DEMs in the second trimester group.

**Additional file 7**.** Table S4**: Function enrichments of DEMs in the third trimester group.

**Additional file 8**.** Table S5**: Pathway enrichments of DEMs in the second trimester group.

**Additional file 9**.** Table S6**: Pathway enrichments of DEMs in the third trimester group.

**Additional file 10**.** Table S7**: Candidate metabolite biomarkers in the second- and third-trimester groups.

## Data Availability

The datasets used or analyzed during the current study are available from the corresponding author (Cheng Hu) on reasonable request.

## Abbreviations

Akt: Protein kinase B
AUC: Area under the curve
BCAAs: Branched-chain amino acids
BMI: Body mass index
DEMs: Differentially expressed metabolites
FPG: Fasting plasma glucose
GDM: Gestational diabetes mellitus
Gpr41: G protein-coupled receptor 41
HDL-C: High-density lipoprotein cholesterol
IRS: Insulin receptor substrate
LDL-C: Low-density lipoprotein cholesterol
LR: Logistic regression
mTOR: Mammalian Target of Rapamycin
mTORC1: Mammalian Target of Rapamycin Complex 1
NGT: Normal glucose tolerance
OGTT: Oral glucose tolerance test
OPLS-DA: Orthogonal partial least square discriminant analysis
PCA: Principal component analysis
PI3K: Phosphatidylinositol-3-kinase
PLS-DA: Partial least square discriminant analysis
RF: Random forest
Rheb: Ras homolog enriched in brain
ROC: Receiver operating characteristic
S6K1: Ribosomal S6 protein kinase 1
SCFAs: Short-chain fatty acids
SMPDB: Small molecule pathway database
TC: Total cholesterol
TG: Total triglyceride
TSC: Tuberous sclerosis complex
UPLC-MS/MS: Ultra-performance liquid chromatography coupled to tandem mass spectrometry
VIP: Variable Important for the Projection
WGCNA: Weighted gene co-expression network analysis
