# Supplementary figures and images for: Discovery of metabolic biomarkers for gestational diabetes mellitus in a Chinese population
Source: Nutr Metab (Lond). 2021 Aug 21;18:79. doi: 10.1186/s12986-021-00606-8 (PMC8379750; doi:10.1186/s12986-021-00606-8)

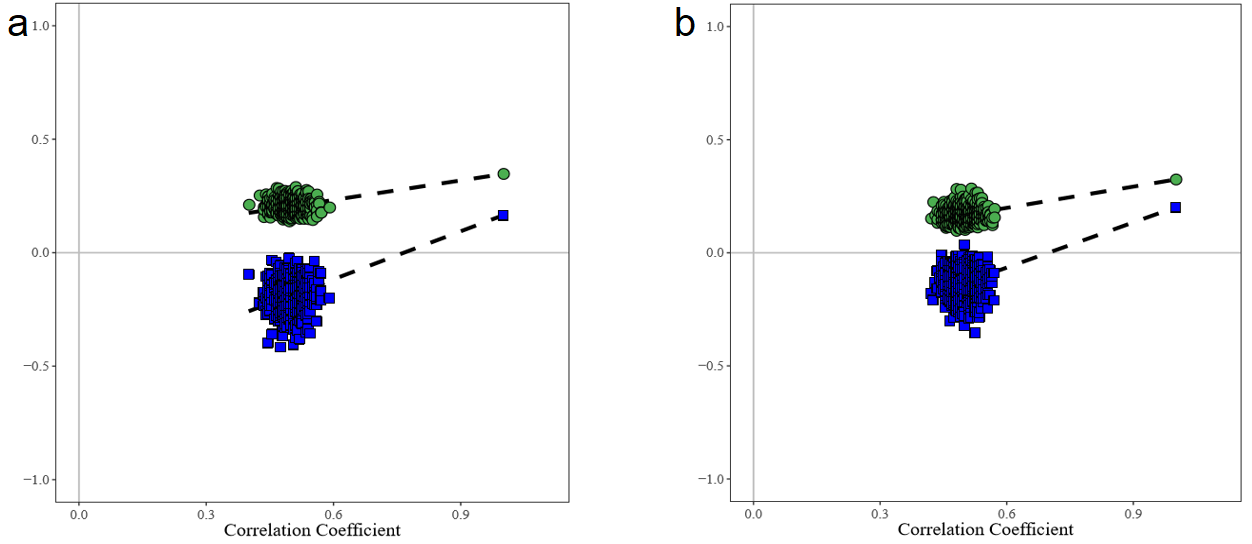

Supplement: Supplementary file 1 — Additional file 1. Figure S1: Permutation test in the second trimester group (a) and the third trimester group (b). [file 12986_2021_606_MOESM1_ESM.tif]

**a**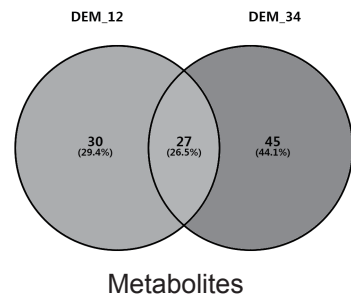**b**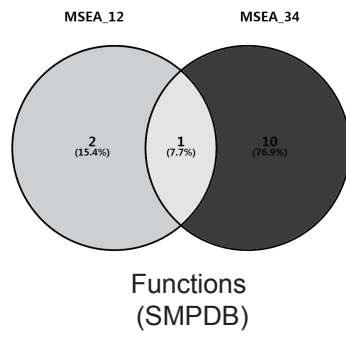**c**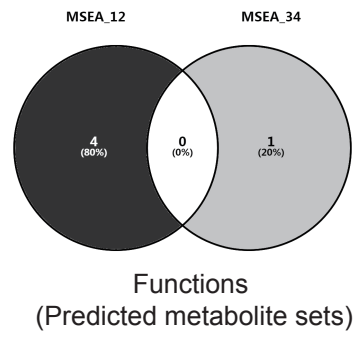**d**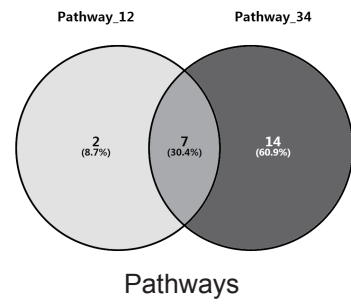

Supplement: Supplementary file 2 — Additional file 2. Figure S2: Overlap statistic of different findings in two trimester groups. [file 12986_2021_606_MOESM2_ESM.pdf]

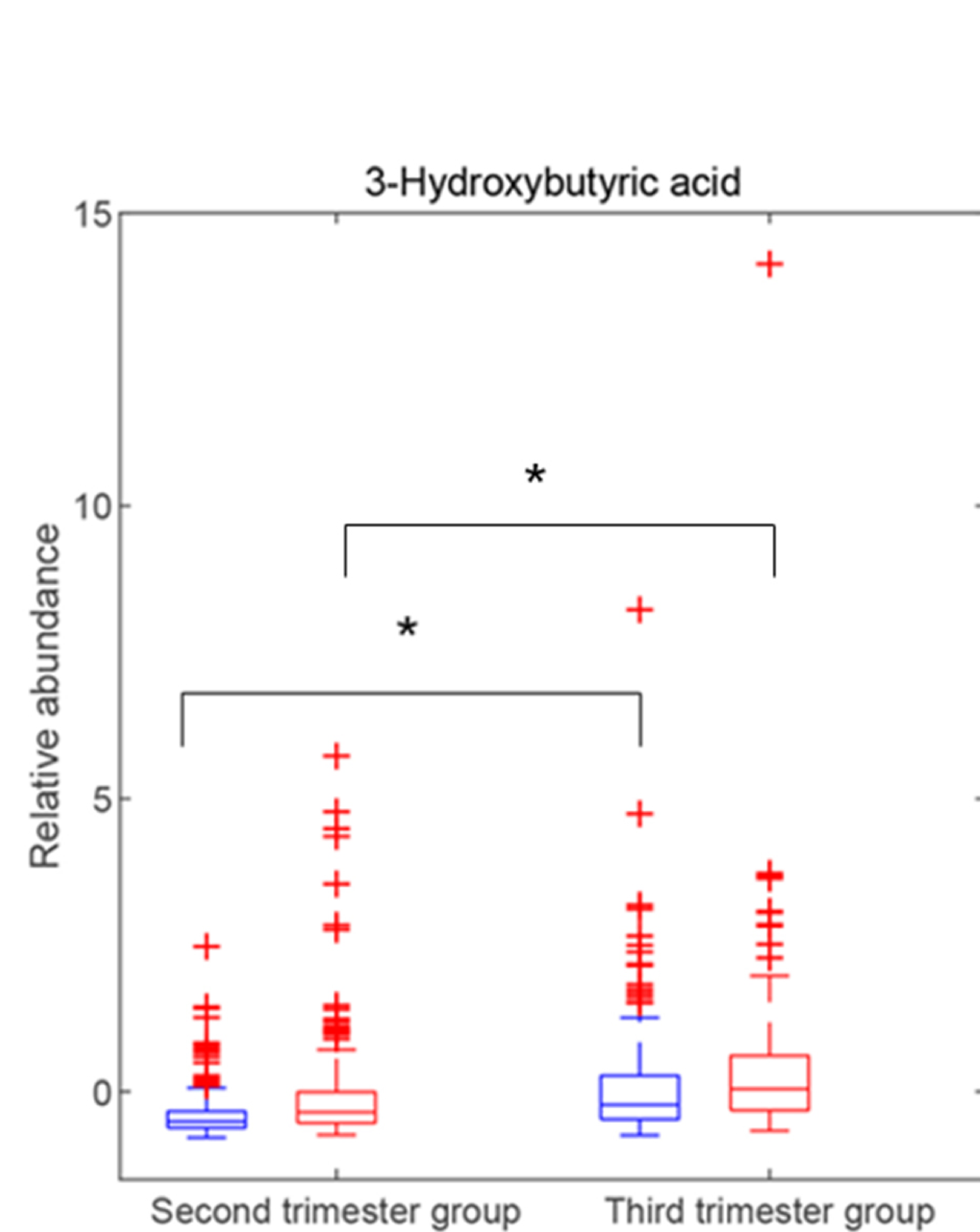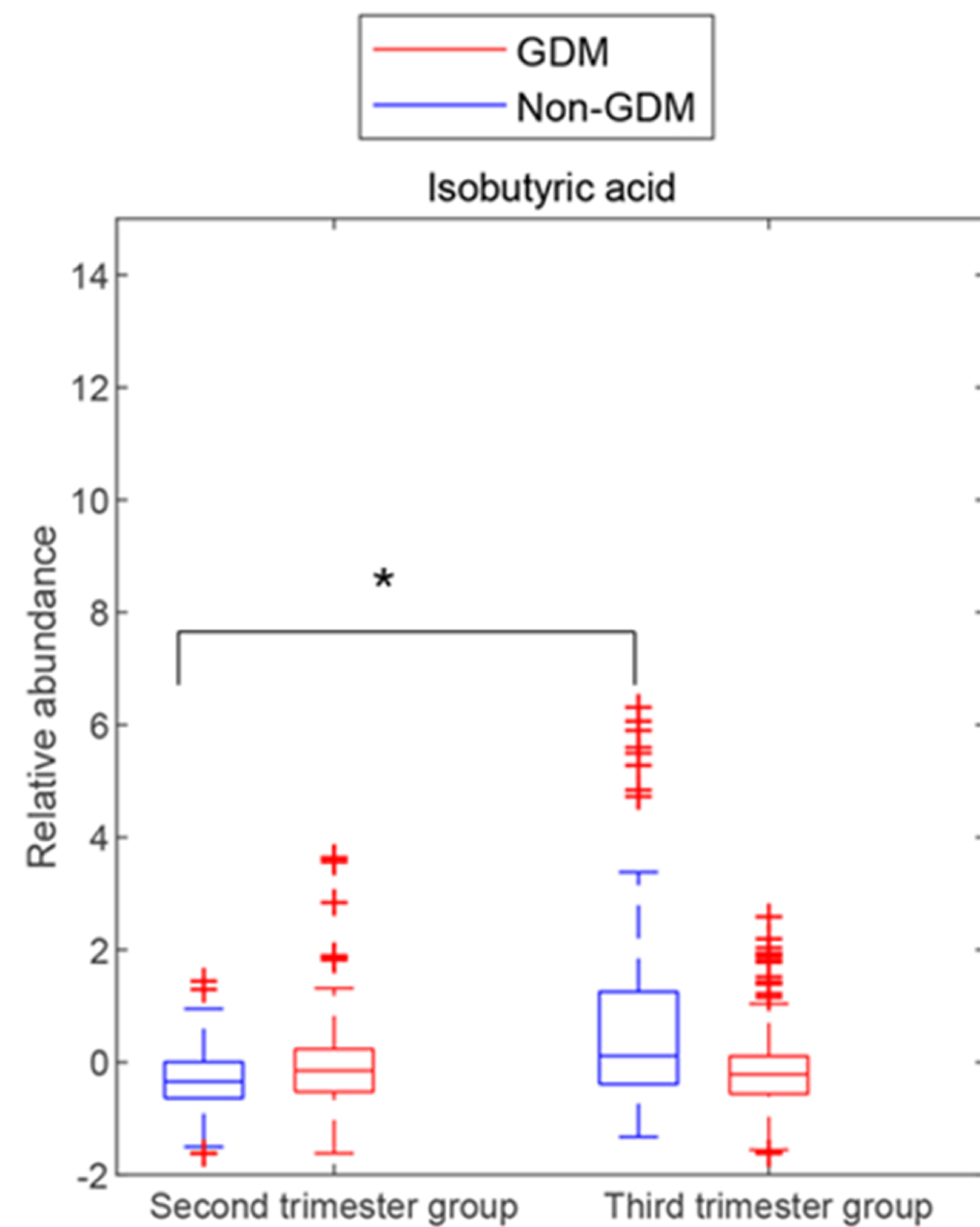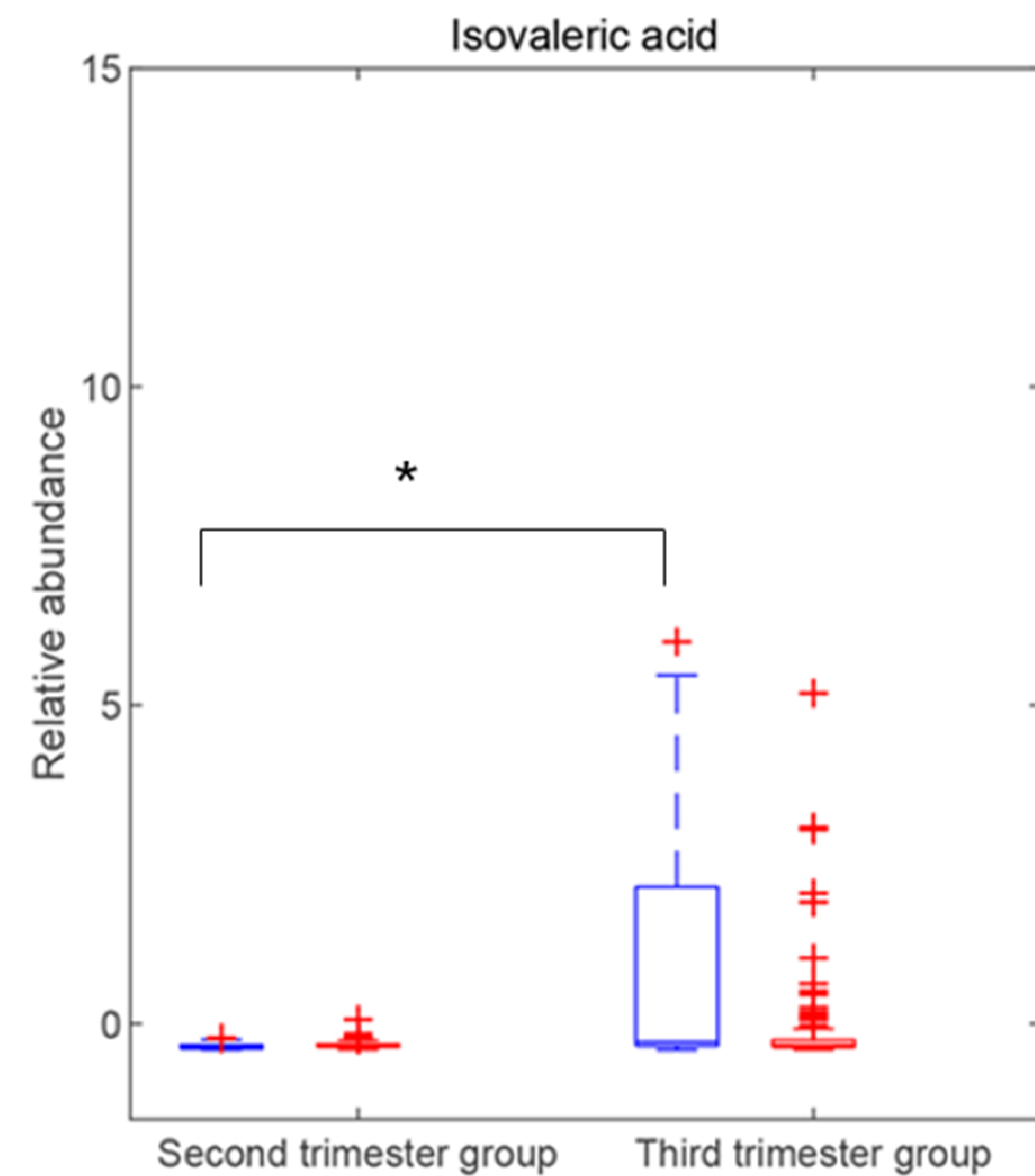

Supplement: Supplementary file 3 — Additional file 3. Figure S3: Longitudinal changes of three potential metabolic biomarkers. *P < 0.05. [file 12986_2021_606_MOESM3_ESM.pdf]
